# Perceived Stress, TikTok Addiction, Self-esteem, and academic performance among university students in Saudi Arabia: A structural path analysis

**DOI:** 10.12669/pjms.42.9.15026

**Published:** 2026-09

**Authors:** Manal Almalki

**Affiliations:** 1 Manal Almalki, BSc, MS, PhDProfessor of Health Informatics, Public Health Department, College of Nursing and Health Sciences, Jazan University, Jazan 45142, Saudi Arabia

**Keywords:** Academic performance, General Strain Theory, Perceived stress, Self-esteem, TikTok addiction, University students

## Abstract

**Background & Objectives:**

TikTok is widely used among university students in Saudi Arabia, raising concerns about its potential role as a maladaptive coping strategy. Limited research has examined how perceived stress, TikTok addiction, and self-esteem jointly influence academic performance within the General Strain Theory (GST) framework. This study investigated the direct and indirect relationships between perceived stress and academic performance, with TikTok addiction and self-esteem as mediating variables.

**Methodology:**

A cross-sectional study was conducted among 400 university students in Saudi Arabia between October and December 2024. Participants completed an online questionnaire that included demographic data, the Perceived Stress Scale (PSS-10), Rosenberg Self-Esteem Scale (RSES), and TikTok Addiction Scale (TTAS). Academic performance was measured using self-reported cumulative grade point average (GPA). After psychometric evaluation of the study instruments, structural path analysis was performed to examine direct and indirect relationships among the study variables.

**Results:**

Participants reported using TikTok for an average of 3.4 hours per day, and 75.3% used TikTok primarily for entertainment. Perceived stress was negatively associated with academic performance (β = -0.12, p < 0.05) and self-esteem (β = -0.11, p < 0.05), while positively associated with TikTok addiction (β = 0.30, p < 0.01). TikTok addiction was negatively associated with academic performance (β = -0.20, p < 0.01), whereas self-esteem was positively associated with academic performance (β = 0.17, p < 0.01). Significant indirect effects were observed through TikTok addiction (β = -0.06, p < 0.01), self-esteem (β = -0.02, p < 0.05), and the sequential pathway involving both mediators (β = -0.01, p < 0.05).

**Conclusion:**

The findings support GST by highlighting that perceived stress influences academic performance through both behavioral and psychological mechanisms. Interventions targeting stress management, healthier TikTok use, and self-esteem enhancement may help improve academic outcomes among university students.

## INTRODUCTION

University students are increasingly exposed to stressors that threaten their psychological well-being and academic success.^1^-^3^ These stressors include coursework overload, examination pressure, and uncertainty about future careers, all of which can impair concentration, motivation, and learning outcomes. Global evidence suggests that over 30–50% of university students experience moderate to high levels of stress.^1^,^2^ In Saudi Arabia, studies have similarly reported that stress-related psychological distress is common among university students and may adversely affect academic engagement and educational outcomes.^4^,^5^

Alongside these challenges, social media platforms have become deeply integrated into students’ daily lives. Among these platforms, TikTok has gained remarkable popularity due to its short-form, highly engaging content.^6^-^8^ Saudi Arabia has one of the highest TikTok penetration rates worldwide.^9^ While TikTok may provide entertainment and temporary relief from stress, concerns have emerged regarding addictive use and its potential effects on mental well-being and academic functioning.^10^-^12^

While stress, social media use, self-esteem, and academic performance have each been examined extensively, the relationships among these factors are often studied in isolation.^10^-^12^ However, less is known about the mechanisms through which perceived stress influences academic performance.^6^-^8^ In particular, little is known about whether addictive TikTok use and self-esteem serve as pathways linking perceived stress to academic outcomes among university students. Understanding these mechanisms is important because it may help identify modifiable targets for interventions aimed at improving student well-being and academic success.^1^-^3^

General Strain Theory (GST) provides a useful perspective for understanding these relationships.^13^ Originally proposed by Agnew, GST suggests that exposure to strain generates negative emotional states that may lead individuals to adopt maladaptive coping behaviors and experience adverse outcomes.^13^ According to this perspective, students experiencing elevated levels of stress may engage in avoidant coping behaviors, such as addictive TikTok use, as a means of managing negative emotions.^14^ Although such behaviors may provide temporary relief, they can also interfere with academic responsibilities and contribute to poorer educational outcomes. At the same time, chronic stress may weaken psychological resources, including self-esteem, which is an important determinant of resilience, motivation, and academic success.^15^

Drawing on GST, the present study conceptualizes perceived stress as the primary strain, TikTok addiction as a maladaptive coping response, self-esteem as a psychological resource affected by strain, and academic performance as the educational outcome. Within this framework, perceived stress may affect academic performance both directly and indirectly through increased TikTok addiction and reduced self-esteem. Furthermore, TikTok addiction may contribute to lower self-esteem, creating a sequential pathway linking stress to poorer academic outcomes. The hypothesized relationships are presented in Fig.1.

**Fig.1:**
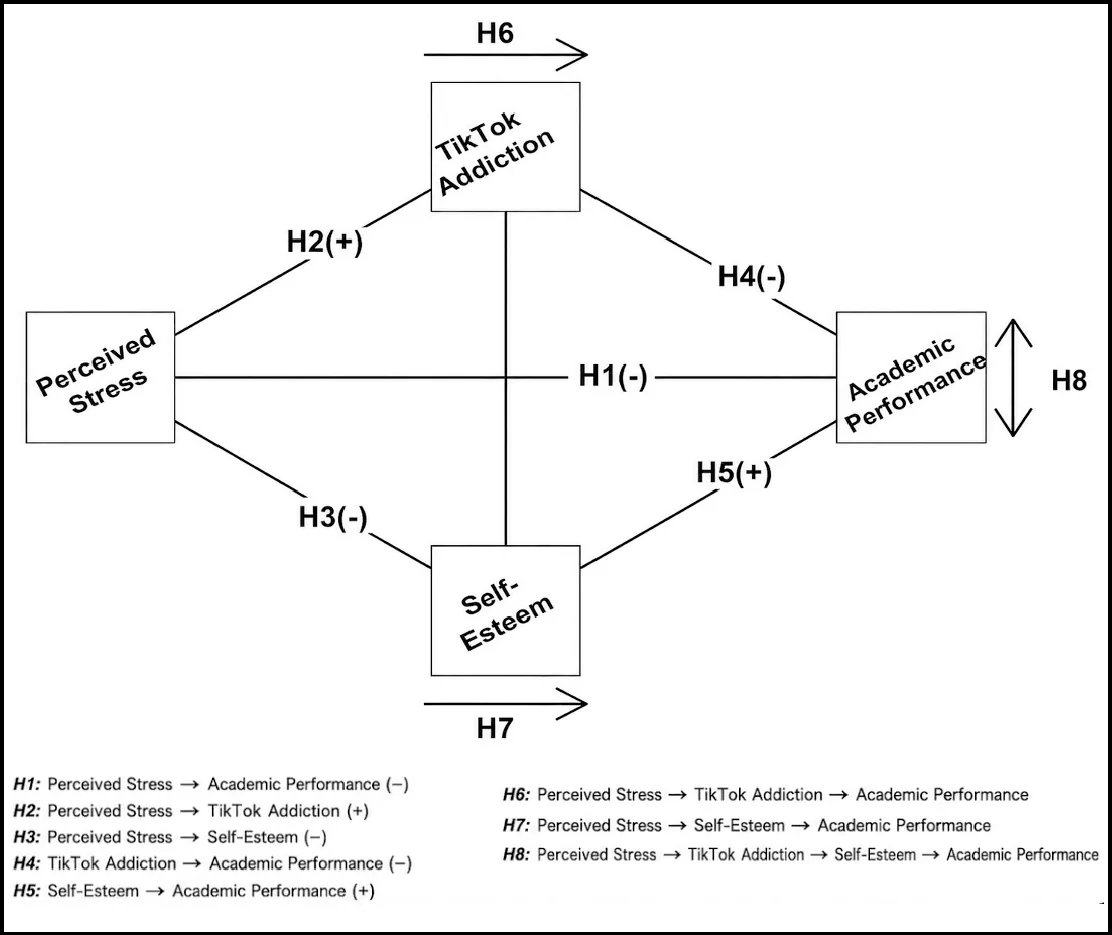
Conceptual model of the hypothesized relationships among perceived stress, TikTok addiction, self-esteem, and academic performance.

Therefore, this study aimed to examine the direct and indirect pathways linking perceived stress to academic performance among university students in Saudi Arabia, with TikTok addiction and self-esteem as mediating variables. By applying GST, this study provides a theory-driven explanation of how psychological strain, digital behaviors, and personal resources jointly influence academic performance.

## METHODOLOGY

A cross-sectional study was conducted between October and December 2024. Eligible participants were undergraduate or postgraduate students enrolled at Saudi universities who provided informed electronic consent.

Participants were recruited using convenience sampling through social networking platforms (i.e., X, TikTok, Telegram, WhatsApp, and Snapchat). The21 study was approved by the Standing Committee for Sabbatical Leaves, Publication and Research Ethics at Jazan University (Ref. No: REC-46/03/1189). Reporting followed the STROBE (Strengthening the Reporting of Observational Studies in Epidemiology) guidelines for cross-sectional studies.^16^

### Measures:

### Perceived Stress:

It was assessed using the 10 items Perceived Stress Scale (PSS-10), which evaluates the extent to which situations in life are perceived as stressful during the previous month.^17^ Items were rated on a 5-point Likert scale. Higher scores indicate greater perceived stress.

### Self-esteem:

It was measured using the 10-item Rosenberg Self-Esteem Scale (RSES), which assesses global self-worth by measuring both positive and negative feelings about the self.^18^ Items were rated on a 4-point Likert scale. Higher scores reflect higher self-esteem.

### TikTok addiction:

It was assessed using the 15-item TikTok Addiction Scale (TTAS), which includes six factors: salience, mood modification, tolerance, withdrawal symptoms, conflict, and relapse.^15^ Items were rated on a 5-point Likert scale. Higher scores indicate higher TikTok addiction.

### Academic performance:

Academic performance was measured using self-reported cumulative grade point average (GPA), categorized as Excellent, Very Good, Good, Pass, and Not Satisfactory according to grading systems used in Saudi Arabia.

### Data collection:

A web-based questionnaire was built using Microsoft Forms, and included sociodemographic characteristics, TikTok usage patterns, and the study measures (Appendix-1). A pilot test involving 11 students was conducted, and minor wording revisions were made to improve clarity. All questionnaire items were mandatory; therefore, no item-level missing data were present in the final dataset.

### Data analysis:

Data were analyzed using R software (Version 4.5.3) within RStudio (Version 2026.05.1; Posit Software, PBC).^19^ Descriptive statistics were calculated as means with standard deviations (SD) for continuous variables, and frequencies with percentages for categorical variables. Descriptive responses to the PSS-10, RSES, and TTAS are presented in Appendix-1.

Before hypothesis testing, the psychometric properties of the PSS-10, RSES, and TTAS were evaluated using confirmatory factor analysis (CFA) with the lavaan package (Version 0.6-21) and the weighted least squares mean and variance adjusted (WLSMV) estimator. Internal consistency was assessed using Cronbach’s alpha, ordinal alpha, and McDonald’s omega, while convergent validity was evaluated using composite reliability (CR) and average variance extracted (AVE). Model fit was assessed using the chi-square statistic (χ²), Comparative Fit Index (CFI), Tucker–Lewis Index (TLI), Root Mean Square Error of Approximation (RMSEA), and Standardized Root Mean Square Residual (SRMR). Reliability and validity analyses were conducted using the semTools (Version 0.5-8) and psych (Version 2.6.5) packages. Detailed psychometric results are presented in Appendix-2. The scales demonstrated acceptable internal consistency, supporting their use in the structural path analysis.

Composite scores for perceived stress, self-esteem, and TikTok addiction were subsequently calculated and entered as observed variables in the structural path analysis (Fig.1). Direct, indirect, and total effects were estimated using maximum likelihood estimation and 5,000 bootstrap samples with bias-corrected 95% confidence intervals. Age, gender, region, economic status, and qualification level were included as covariates.

The final sample size (N=400) was considered adequate for both the psychometric validation and structural path analysis. For the CFA, the study included 35 observed indicators across the PSS-10, RSES, and TTAS, yielding a cases-per-indicator ratio of 11.4:1, which exceeds the commonly recommended guideline of 10:1.^20^,^21^ The sample size also exceeded commonly recommended minimums for structural path analysis using observed variables, providing sufficient precision to estimate the hypothesized direct and indirect effects.^20^,^21^ Statistical significance was set at p < 0.05.

## RESULTS

### Participants’ Demographic and Academic Characteristics:

A total of 417 questionnaires were received. Seventeen responses were excluded because informed consent was not provided, resulting in a final sample of 400 students. Most participants were female (81.5%), aged 18–24 years (77.8%), and studying in the southern region of Saudi Arabia (78.3%). More than half reported very good health status (62.3%), were enrolled in a bachelor’s degree program (73.3%), and reported excellent cumulative GPA scores (56.8%). Nearly half were in the final stage of their academic program (49.5%), while 47.5% were enrolled in health or medical related majors (Table-I).

**Table-I:** Demographic and academic characteristics.

Characteristics	Value	n(%)
Gender	Female	326(81.5%)
	Male	74(18.5%)
Age	18-24 years	311(77.8%)
	25-34 years	63(15.8%)
	≥35 years	26(6.5%)
Region (where you study)	South (Jazan, Asir, Al-Baha, Najran)	313(78.3%)
	North (Tabuk, Northern Borders, Al-Jawf)	11(2.8%)
	Middle (Riyadh, Qassim, Hail)	34(8.5%)
	West (Mecca, Medina)	27(6.8%)
	East (Eastern Region)	15(3.8%)
Current health status	Very good	249(62.3%)
	Good	126(31.5%)
	Moderate	23(5.8%)
	Poor	2(0.5%)
Economic status	Good	189(47.3%)
	Moderate	188(47.0%)
	Poor	23(5.8%)
Current Qualification Level	Diploma, or associate degree	65(16.3%)
	Bachelor’s degree or equivalent	293(73.3%)
	Higher diploma or equivalent	21(5.3%)
	Master’s degree or equivalent	12(3.0%)
	PhD or equivalent	9(2.3%)
Main major	Health or medical-related major	190(47.5%)
	Science, Engineering, or Computer-related major	98(24.5%)
	Law, Arts, Linguistics, or Humanities-related major	45(11.3%)
	Business and Management-related major	67(16.8%)
Years in current study program	Early stage – Just started the program or in the first year.	103(25.8%)
	Midway – In the middle of the study program, about halfway through.	99(24.8%)
	Near completion – Approaching the end of the program or preparing for graduation soon.	198(49.5%)
GPA	Excellent: [4.50-5.00/5] or [3.50-4.00/4] or 90% and above	227(56.8%)
	Very Good: [3.75-4.49/5] or [2.75 or 2.75-3.49/4] or 80-89%	111(27.8%)
	Good: [2.75-3.74/5] or [1.75-2.74/4] or 70-79%	44(11.0%)
	Pass: [2.00-2.74/5] or [1.00-1.74/4] or 60-69%	14(3.5%)
	Not satisfactory: [Below 2.00/5] or [Below 1.00/4] or 59% and below	4(1.0%)

### TikTok Usage Patterns:

More than half of the students reported using a fake name on their TikTok accounts (54.5%), and 88.8% had 1,000 or fewer followers. The majority (57.3%) had been using TikTok for more than two years, primarily for entertainment purposes (75.3%). The average daily TikTok usage was 3.4 hours (SD = 2.1) (Table-II).

**Table-II:** TikTok usage characteristics.

Variable	Value	n(%), Mean(SD)*
TikTok account name	Fake name	218(54.5%)
	Real name	182(45.5%)
TikTok followers	1K followers or less	355(88.8%)
	More than 1K followers	45(11.3%)
Duration of TikTok use	≤2 years	171(42.8%)
	>2 years	229(57.3%)
Daily TikTok use (hours)	Number of hours [1 to 10]	3.4 (2.1)*
Main purpose of using TikTok	Education & learning	81(20.3%)
	Entertainment	301(75.3%)
	Promoting/doing a business	18(4.5%)

### Structural Path Analysis:

The structural path analysis supported all hypothesized relationships (H1 to H8) (Table-III and Fig.2). Perceived stress was negatively associated with academic performance (β = -0.12, p < 0.05) and self-esteem (β = -0.11, p < 0.05), while positively associated with TikTok addiction (β = 0.30, p < 0.01). In turn, TikTok addiction was negatively associated with academic performance (β = -0.20, p < 0.01), whereas self-esteem was positively associated with academic performance (β = 0.17, p < 0.01).

**Table-III:** Direct and indirect effects of perceived stress, TikTok addiction, and self-esteem on academic performance.

Hypotheses	B	β	SE	95% CI	P-Value	Decision
Direct Effect						
H1: Perceived stress is negatively associated with academic performance (GPA). Perceived Stress → Academic Performance.	-0.18	-0.12	0.05	[-0.22,-0.01]	< 0.05*	Accepted
H2: Perceived stress is positively associated with TikTok addiction. Perceived Stress → TikTok addiction.	0.25	0.30	0.04	[0.22,0.37]	< 0.01**	Accepted
H3: Perceived stress is negatively associated with self-esteem. Perceived Stress → Self-esteem.	-0.12	-0.11	0.06	[-0.22,0.00]	< 0.05*	Accepted
H4: TikTok addiction is negatively associated with academic performance. TikTok addiction → Academic Performance.	-0.36	-0.20	0.06	[-0.31,-0.07]	< 0.01**	Accepted
H5: Self-esteem is positively associated with academic performance. Self-esteem → Academic Performance.	0.24	0.17	0.05	[0.06,0.27]	< 0.01**	Accepted
Indirect Effect						
H6: TikTok addiction mediates the relationship between perceived stress and academic performance. Perceived Stress → TikTok Addiction → Academic Performance.	-0.09	-0.06	0.02	[-0.10,-0.02]	< 0.01**	Accepted
H7: Self-esteem mediates the relationship between perceived stress and academic performance. Perceived Stress → Self-Esteem → Academic Performance.	-0.03	-0.02	0.01	[-0.04,0.00]	< 0.05*	Accepted
H8: TikTok addiction and self-esteem sequentially mediate the relationship between perceived stress and academic performance. Perceived Stress → TikTok Addiction → Self-Esteem → Academic Performance.	-0.02	-0.01	0.01	[-0.02,-0.00]	< 0.05*	Accepted
Total Indirect Effect	-0.14	-0.09	0.02	[-0.13,-0.04]	< 0.01**	
Total Effect	-0.31	-0.20	0.05	[-0.30,-0.10]	< 0.01**	

B = Unstandardized coefficient; β = Standardized coefficient; CI = 95% Confidence interval; SE = Standard error;

** p<0.01

* p<0.05. Age, gender, region, economic status, and qualification level were included as covariates.

**Fig.2:**
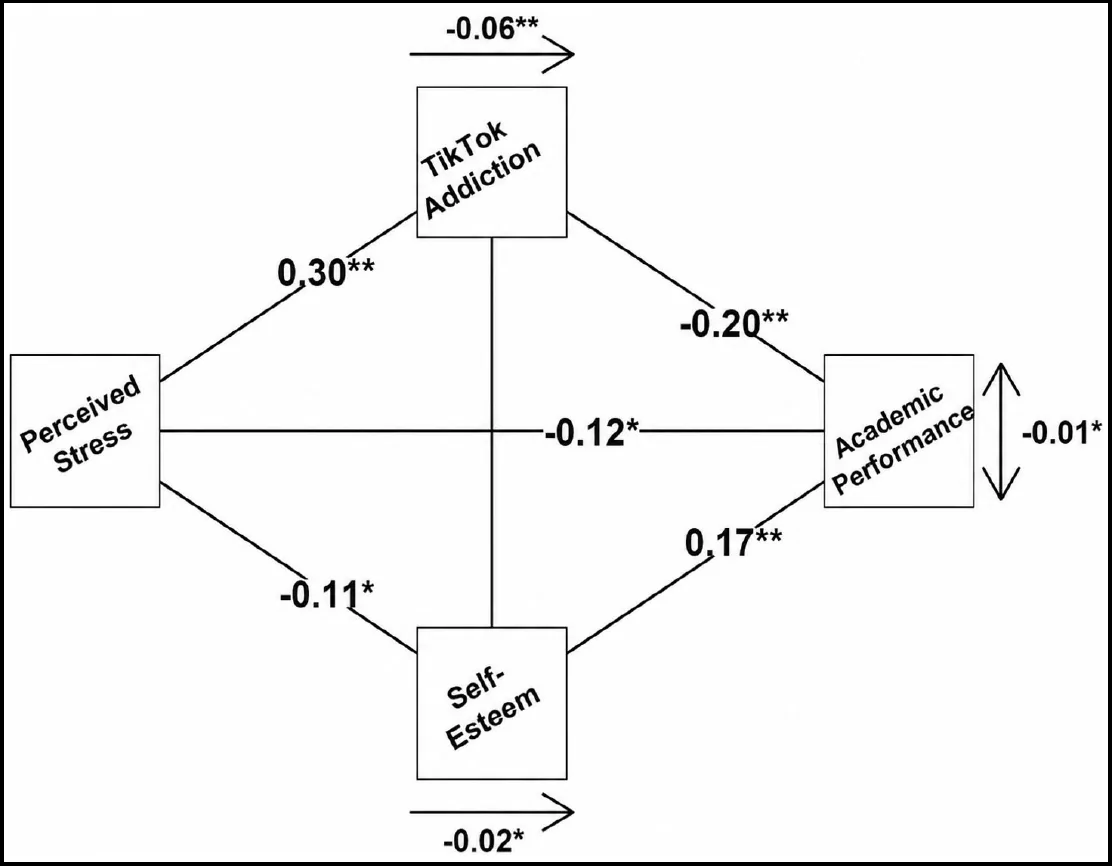
Structural path analysis. Values represent standardized path coefficients (β). **p<0.01; *p<0.05.

Significant indirect effects were observed through TikTok addiction (β = -0.06, p < 0.01), self-esteem (β = -0.02, p < 0.05), and their sequential mediation pathway (β = -0.01, p < 0.05). The total indirect effect remained significant (β = -0.09, p < 0.01), and the overall effect of perceived stress on academic performance was negative (β = -0.20, p < 0.01). The structural path model demonstrated excellent fit (χ² = 10.04, df = 10, p = 0.437, CFI = 0.998, TLI = 0.999, RMSEA = 0.003, SRMR < 0.001).

## DISCUSSION

This study investigated the relationships among perceived stress, TikTok addiction, self-esteem, and academic performance among university students in Saudi Arabia using the GST framework. All hypothesized relationships were supported, indicating that perceived stress influences academic performance through both direct and indirect pathways involving TikTok addiction and self-esteem.

### Perceived Stress, TikTok Addiction, and Academic Performance (H1, H2 & H4):

Perceived stress was negatively associated with academic performance and positively associated with TikTok addiction. These findings are consistent with previous studies showing that stress impairs academic functioning while increasing vulnerability to addictive TikTok use.^10^-^12^ From a GST perspective, stress represents a form of strain that encourages avoidant coping behaviors.^13^ Students may turn to TikTok as a means of temporarily relieving negative emotions; however, excessive use may reduce study time, impair concentration, and interfere with academic responsibilities, ultimately contributing to poorer academic performance. 2,10-12

### Perceived Stress, Self-Esteem, and Academic Performance (H3 & H5):

Perceived stress was also negatively associated with self-esteem, whereas self-esteem was positively associated with academic performance. These findings suggest that higher levels of stress may erode students’ confidence and self-worth, reducing academic motivation, resilience, and engagement in academic activities.^2^,^11^,^12^ Within the GST perspective, self-esteem represents an important psychological resource that can be weakened by prolonged strain, thereby increasing vulnerability to poorer academic outcomes.^15^,^11^,^22^

### Mediation and Sequential Mediation Effects (H6–H8):

A key contribution of this study is the identification of both independent and sequential mediation pathways. TikTok addiction and self-esteem each independently mediated the relationship between perceived stress and academic performance, indicating that stress influences academic outcomes through both behavioral and psychological mechanisms. Furthermore, the significant sequential pathway suggests that greater perceived stress may increase TikTok addiction, which may subsequently reduce self-esteem and contribute to poorer academic performance. By integrating these variables within a single theory-driven model, the findings extend previous research and provide empirical support for GST’s proposition that strain may trigger maladaptive coping behaviors that ultimately undermine positive functioning and achievement.^8^,^13^

### Implications:

The findings have practical implications for universities and student-support services. Interventions that reduce perceived stress may improve both psychological well-being and academic achievement. Stress management programs, counseling services, mindfulness training, and time-management interventions may help students adopt healthier coping strategies.^3^-^8^ In addition, digital well-being initiatives that promote responsible TikTok use, together with programs that strengthen self-esteem and psychological resilience, may further support academic success.^1^-^3^,^12^

### Strengths and Limitations:

A major strength of this study is the application of a theory-driven framework to examine the direct and indirect pathways linking perceived stress, TikTok addiction, self-esteem, and academic performance. However, the cross-sectional design impedes causal conclusions,^23^ and the reliance on self-reported measures may have introduced social desirability or recall bias.^24^ Future longitudinal studies are needed to examine how perceived stress, TikTok use, self-esteem, and academic performance evolve over time.^8^,^10^

## CONCLUSION

This study provides evidence that perceived stress influences academic performance among university students in Saudi Arabia through both direct and indirect pathways. Consistent with GST, increased TikTok addiction and reduced self-esteem partially explained the association between perceived stress and academic performance, including a significant sequential mediation pathway. These findings highlight the importance of integrated interventions that reduce stress, promote healthier TikTok use, and strengthen self-esteem to support student academic success. Future longitudinal research is needed to confirm these pathways and further examine the role of digital behaviors in academic achievement.
